# Mechanical behaviour of alginate film with embedded voids under compression-decompression cycles

**DOI:** 10.1038/s41598-019-49589-w

**Published:** 2019-09-13

**Authors:** Arindam Banerjee, Somenath Ganguly

**Affiliations:** Department of Chemical Engineering, Indian Institute of Technology, Kharagpur, 721302 India

**Keywords:** Biomedical engineering, Chemical engineering, Gels and hydrogels

## Abstract

Voids of 300 µm diameter were embedded uniformly as monolayer in alginate gel film using a fluidic device. Voids of these dimensions in biopolymer gel film are desired for better transport of bioactive species and cell colonization in engineered tissues. In this article, the role of embedded voids in reducing compressive stress, hysteresis, and time scale of reheal vis-a-vis expulsion of pore fluid and its reabsorption upon reversal of load are reviewed. The cyclic loading was conducted with varying amplitude and frequency. The irreversible changes, if any in the gel structure under extreme compression were analyzed. The rate of expulsion of aqueous phase directly relates to the permeability of the gel film that is estimated here using simplified momentum and volumetric balance equations. The decrease in permeability with deformation is analyzed further, and the contribution of voids in this regard is discussed.

## Introduction

Hydrogels are soft and porous material with large volume of trapped water in the three dimensional polymer networks. Unique properties of hydrogel are found suitable for many applications e.g., drug delivery, tissue reconstruction, water treatment, and making of superabsorbents and sensors^1–6^. Hydrogels, based on naturally available biopolymers are frequently used in biomedical devices because of their favourable mechanical characteristics, pore network for easy transport of fluids, biocompatibility, and the ease of degradation in body fluid. The compression and decompression, either monotonous, or under cyclic loading may cause flow of solution through the gel network. Such flow is critical in transport of bioactive species, and also cushioning against *in vivo* stresses. In particular, the hydrogel structure is meant to withstand tensile, compressive and shear loadings arising from the movement in muscles, and also tissue around blood vessels. These forces lead to the expulsion of aqueous phase from the gel matrix. The expelled fluid may act as lubricant around the faces of contact. Upon removal of these forces, the gel matrix may absorb the expelled fluid, and return to its original state. This loss and the subsequent gain of fluid by the gel matrix depend upon the magnitude of deformation, the rate of deformation, and also the relaxation rate of the hydrogel network at microscale^7^.

Many research groups^8–11^ have studied the compressive loading on biomaterials, bones and cartilages. Ferguson *et al*.^10^ performed compression-decompression cycle on intervertebral disc, where 11% of total height was lost during compression over a period of 16 hours. This entire lost height was regained over 8 hours of swelling. Growney Kalaf *et al*.^8^ performed cyclic loading on alginate with amplitude of deformation as 20%. This was reported at regular intervals over a period of 28 days with an object to study hysteresis, and suitability of alginate for intervertebral disc tissue engineering. Goffman and Buyonor^11^ performed cyclic compression on swollen cellulose-polyacrylamide hydrogel composite, and measured hysteresis at varying deformation rates. They used 30% to 50% as amplitude of deformation with the understanding that these values correspond to the maximum compression that a human knee cartilage is subjected to under various types of body movement. Kluge *et al*.^12^ applied the theory of porous media consolidation, as applied to problems of geomechanics to explain confined compression creep test results for silk hydrogel. Cai *et al*.^13^ employed a poroelastic model to analyze relaxation test results for alginate gel, implicitly accounting the transport of aqueous phase in the gel matrix. Urciuolo *et al*.^14^ considered compression, Poiseuille’s flow and solute release together, assuming functional forms for permeability and diffusion parameters, based on prior studies of cartilages (Ateshian *et al*.^15^, Evans and Quinn^16^). Zhang *et al*.^17^ measured the expelled volume for poly vinyl alcohol (PVA) gel with an object to relate the stress response with the water content of gel.

The performance of a hydrogel can be improved by inserting voids in the gel matrix at the time of fabrication^18,19^. There are different methods to embed voids within polymer matrix, such as emulsion freeze drying, solvent casting or particulate leaching, fibre bonding, thermal phase separation, electrospinning, gas foaming, and through use of supercritical CO_2_^20^. In more recent years, fluidic devices were leveraged to generate mono-disperse bubbles that align on their own into uniform array^21–26^. In the latter method, the gel is not subjected to thermal or chemical treatment. Also, the process does not involve costly apparatus, as required for free-form-fabrication.

Hydrogel may be considered as a network of physical crosslinks, saturated with water. The crosslinked network provides intrinsic porosity that has been used extensively in many applications. In this study, macrovoids of diameter on the order of 300 µm was embedded uniformly over the entire gel structure using a fluidic device. The pores, thus developed over and above the intrinsic pores of the crosslinked network are referred as macropores in this article. The macropores of such dimensions are known to help in cell colonization, and also in drug delivery applications. Here, it is further evaluated how the embedded macrovoids influence the stress-response, when the gel structure is subjected to compressive load. Both types of pores are fully saturated with PBS buffer, simulating body fluid in this work. Alginate is considered in this experimentation as the candidate gel matrix. Alginate, a polysaccharide is extracted from brown algae, and is known for its application in drug delivery and tissue engineering^27–30^. The compressive load on the top surface of the gel structure has to be supported by both the solid skeleton of crosslinked network, and the water in the pores. The total stress is composed of (i) the effective stress that imposes on the gel skeleton, and (ii) the pore water pressure. The latter component may equilibrate across the gel structure through pressure-driven flow that finally exudes from the surface. The effective stress on the solid skeleton relaxes with time due to viscoelastic character of the crosslinked network. The work plan here is to impose compression on fully swelled alginate film with embedded macrovoids, and study the stress-strain behaviour, water expulsion, internal structure (SEM images), and permanent change in dimensions, if any. The exercise is extended to decompression with consequent absorption of water, and study of residual stress. Finally, the effect of stress cycles with varied amplitude and frequency is reviewed.

## Results and Discussions

**Figure 1:**
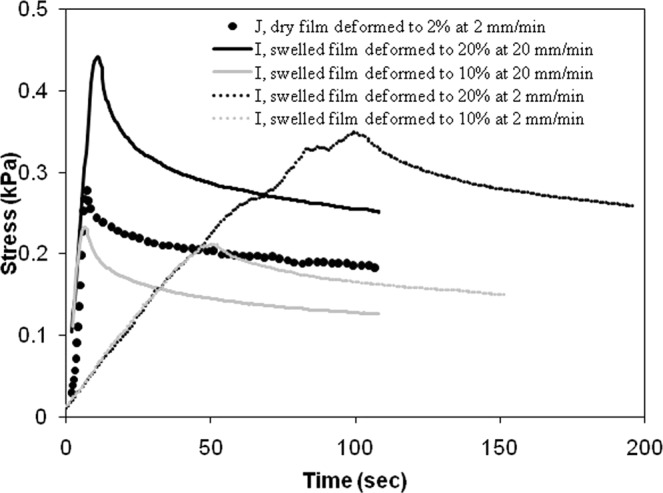
Relaxation test on dry and swelled gel films (tags I and J) without voids.

Alginate gel is viscoelastic^29,31,32^ and supposedly shows non-linear stress-strain behaviour. Further, the compression leads to build-up of pore pressure for the water, trapped inside the gel network. The water has to overcome the hydraulic resistance to initiate a Poiseuille’s flow and ooze out of the gel matrix, until the pore pressure equilibrates with the surrounding pressure. This equilibration process, which has a different time scale, compared to that of viscoelastic relaxation contributes to the non-linearity in stress-strain plot. Figure 1 shows the stress relaxation for dry and swelled gel films respectively. In case of dry alginate gel, the pore pressure does not arise, and the viscoelasticity of the solid skeleton alone determines the time period for stress relaxation. The effect of deformation rate on the peak stress and the time period for stress relaxation are demonstrated in this figure. For the swelled gel film, the peak stress was much lower, and it took much longer for the stress to equilibrate, when compared with behaviour of dry gel film.

**Figure 2:**
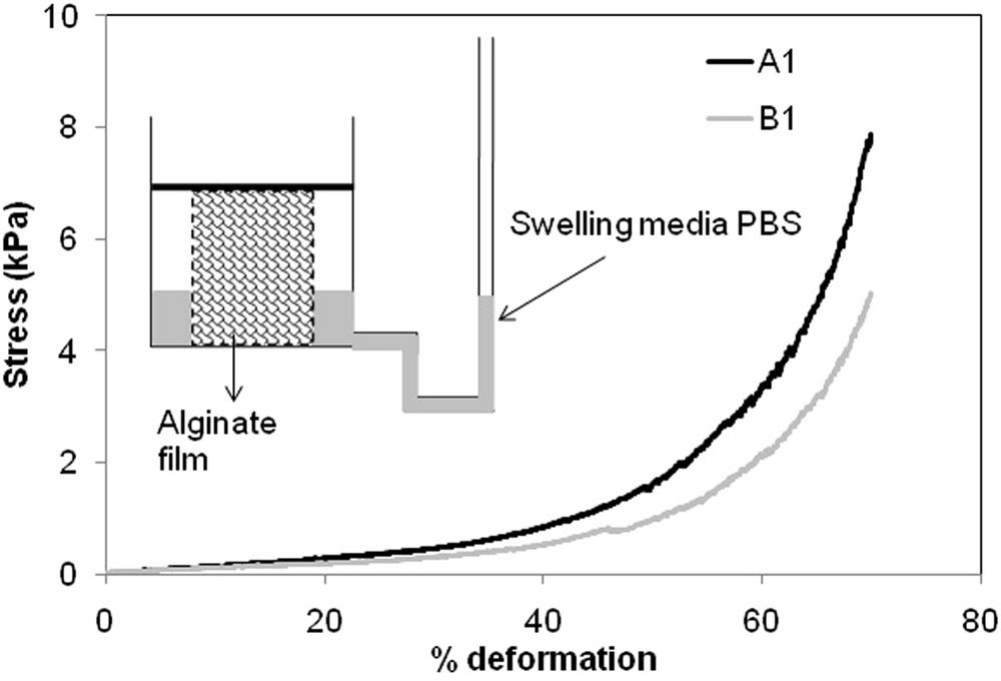
Stress responses at a deformation rate of 2 mm/min.

With this stress-relaxation at the backdrop, the primary objective here is to study the effect of embedded voids in the alginate gel structure under two different forms of compressive loading. Figure 2 shows the stress response, where compressive load was monotonously increased at a constant deformation rate. The compressive stress was reduced due to the presence of voids. This may be hypothesized that the macrovoids provided sites for lateral bulging, and release of pore pressure through easier flow of aqueous phase through the void network.

**Figure 3:**
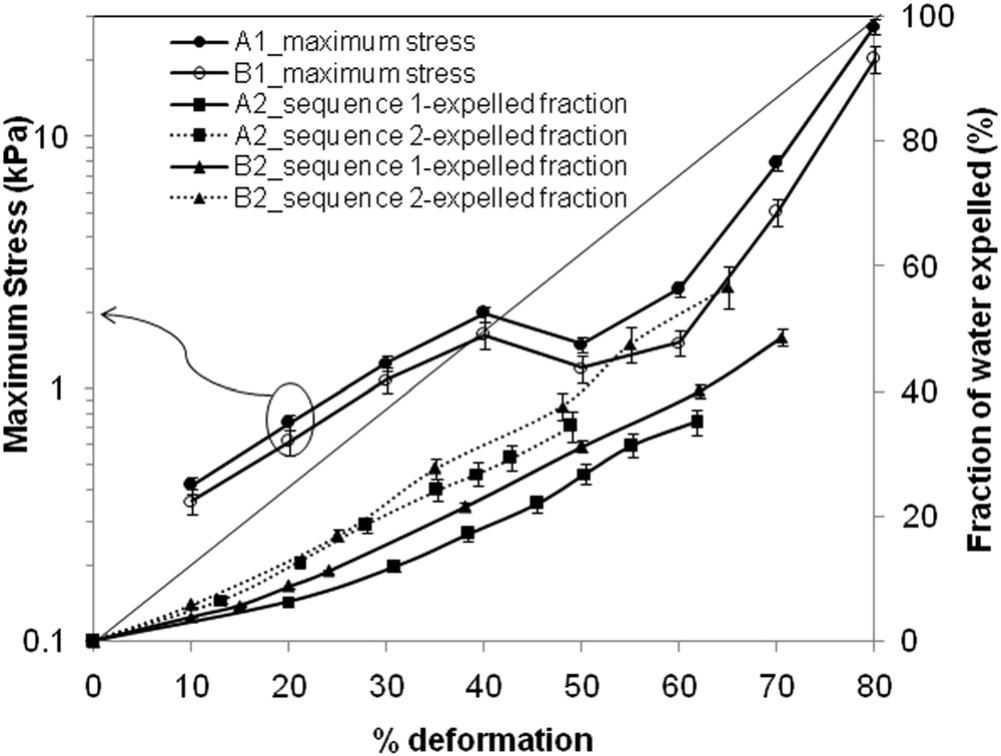
Peak stress and extent of expulsion at different deformation level.

The alginate gel showed unique ability to reabsorb the expelled aqueous phase, and subsequently respond to the compressive strain in a very similar way, as the freshly prepared gel deforming for the first time. Here, the gel structure was compressed phase wise, with decompression, and reswelling at the end of each phase. The reswelling was performed by dipping the gel sample in PBS buffer over night at room temperature. The end-point stress for each compression stage is plotted following the primary axis in Fig. 3. The y-axis is in log-scale, and the non-linearity is consistent with the previous figure. For comparison, two gel films (with tags A2 and B2 in Table 1) were directly subjected to 80% deformation without any prior compression (Test 2 in Fig. S2 of Supplementary File). One of these two films contained voids. The same deformation rate of 2 mm/min was maintained. The experiments were done thrice and the error ranging from 2–7% is shown in the plot. The stress at 80% deformation was found to be 12 kPa and 8 kPa for film without void and for film with void respectively. These values are somewhat lower than the corresponding stress values at 80% deformation after stage-wise compression (Fig. 3). Test 2 was continued for the same set of films decompression, reswelling in PBS buffer, and recompression to 80% deformation in the same manner as before. At this stage, the stress at 80% deformation was found to be 49.6 kPa and 38 kPa for the film without void and for the film with void respectively. These values are even higher than the corresponding maximum stress after stage-wise compression in Fig. 3. That is, the change in mechanical characteristics of gel due to such extreme level of compression is not significant. However, the compressive stress in the film with voids was always found less, compared to similar film without voids.

**Table 1:** Descriptions of alginate films.

Films tag	Diameter (mm)	Thickness (mm)	Presence of voids
A1 to A4	42 ± 2.21	22 ± 0.24	Without void
I	32.6 ± 1.04	17.5 ± 0.12	
J	36 ± 1.51	9.1 ± 0.42	
K	37.16 ± 2.3	18.46 ± 0.08	
B1 to B4	41 ± 3.15	23 ± 0.37	With voids

The secondary axis in Fig. 3 refers to the release of aqueous phase from the gel film at different levels of deformation, when subjected to monotonous compression. The volume of expelled phase linearly increased with the extent of deformation. The presence of voids resulted in expulsion of greater volume. Understandably, the aqueous phase held inside the voids are more mobile under a given compressive stress, compared to the aqueous phase, trapped in the crosslinked network. The fractional volume of aqueous phase, expelled at each level of compression was substantially less than the fraction of initial thickness, squeezed at that point. This is suggestive of lateral bulging in the gel structure, and also the finite time scale in release of pore pressure. The expulsion of aqueous phase after one round of compression-decompression cycle was faster due to the availability of already established pathways. In a separate experiment, the calcium content in the expelled aqueous phase was estimated to ascertain the extent of leaching and/or erosion in gel structure. The calcium concentration in the original gel sample was found to be 4.7 wt%, whereas the calcium concentration in the exuded fluid dropped from 0.44 wt% to 0.28 wt%, even after the level of deformation was raised to 80%. This may be noted that the alginate gel releases calcium ion in exchange of sodium ion in the PBS buffer, thereby degrading the gel network within a few days^33,34^. Therefore, within the time frame of compression experiments and repeated overnight swelling, the release of calcium ion at a concentration level of 5–6% due to degradation is very much anticipated.

The rate of expulsion of aqueous phase is governed by the gradient in pore pressure, which is related to excess stress, imposed by the compression platen. The permeability of the gel structure at different stages of compression is estimated based on a momentum balance equation in cylindrical coordinates (Eq. 1), assuming azimuthal symmetry (σ_θ_ = 0).

1 $$\frac{\partial {\sigma ^{\prime} }_{r}}{\partial r}+\frac{{\sigma ^{\prime} }_{r}}{r}=\frac{\partial P}{\partial r}$$

Further, the rate of radial flow out from a cylindrical element around any radial position ***r*** due to longitudinal deformation at a rate ***w*** is written in Eq. 2, assuming pseudo-steady state.

2 $$\pi {r}^{2}w=-\,\frac{k}{\mu }\frac{\partial P}{\partial r}(2\pi rh)$$

Here, *h* = thickness of gel layer

*w* = rate of longitudinal deformation (in z-direction), equivalent to the rate of expulsion from the sides

*k* = permeability of the gel film

*μ* = viscosity of the aqueous phase

σ = excess stress

z = vertical axis in the direction of compression

r = radial axis

Eliminating $\partial P/\partial r$ from the two equations,

3 $$\frac{\partial {\sigma ^{\prime} }_{r}}{\partial r}+\frac{{\sigma ^{\prime} }_{r}}{r}=-\,\frac{\mu rw}{2kh}$$

Upon integration,

4 $${\sigma ^{\prime} }_{r}=\frac{1}{r}[\int {C}_{1}\frac{{r}^{3}}{3}+C]$$

Here, *C* is the constant of integration, and may be evaluated using ${\sigma ^{\prime} }_{r}(at\,r=R)=0$

*C*_*1*_ = −*µw*/*2* *kh*

γ = Poisson’s ratio

Also,

5 $${\sigma ^{\prime} }_{zz}=\gamma {\sigma ^{\prime} }_{rr}$$

Therefore, the overall stress, imposed by the compression platen ${\bar{\sigma }^{\prime} }_{zz}$ can be written as

6 $${\bar{\sigma }^{\prime} }_{zz}=\frac{\gamma }{\pi {R}^{2}}{\int }_{0}^{R}\frac{1}{r}[{C}_{1}\frac{{r}^{3}}{3}+C]2\pi r\partial r$$

Upon integration of Eq. (6),

7 $${\bar{\sigma }^{\prime} }_{zz}=\frac{\gamma {R}^{2}\mu w}{4\,kh}$$

**Figure 4:**
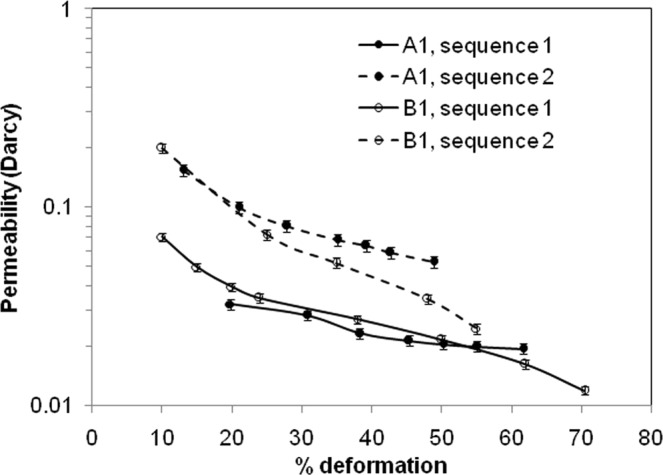
Change in permeability of the alginate gel film during compression.

During compression, the ${\bar{\sigma }^{\prime} }_{{\bf{zz}}}\,$ was measured as stress, and ***h*** could be obtained from the corresponding strain measurement. ***w*** was obtained from the experimental data on expulsion rate (***Q***). Permeability, ***k*** is the only unknown in the above expression, and can be estimated at different extents of deformation. The expulsion rate, ***Q*** was estimated by fitting a second order polynomial to the cumulative flow vs. time plot, and taking derivative of the polynomial expression with time. The R^2^ fit of the polynomial expression was 0.98 ± 0.02. The experiments were done thrice and the error ranging from 0.5–2% is shown in the plot. In the initial stage of compression, the permeability (Fig. 4) was found to be somewhat higher for films containing voids. Understandably, the voids provided additional conduit for flow through the structure. This may be noted that the regular ordering of voids enables faster movement of aqueous phase through entire matrix. A set of irregular voids would have resulted in the larger ones either getting connected, forming channels within the gel matrix, or the larger ones existing as isolated stagnant zones that do not contribute much to the flow of aqueous phase. Further, the permeability got reduced with deformation. This was due to compaction of the gel strands that constricted the expulsion pathways. Also, the difference in permeability due to the presence of voids no longer existed at higher level of deformation. This implies that the voids were fully compressed at higher level of deformation, and did not provide any additional pathways. The gel film after extreme compression and overnight swelling was subjected to a second phase of compression. There was an increase in permeability in this second phase. The skeleton of the gel was already compromised during the first phase of compression. This was manifested in more open pathways for Poiseuille’s flow. However, decrease in permeability due to deformation is still evident. Typical permeability value reported in the literature are 0.0436 × 10^−3^ to 669.75 Darcy for agarose gel^14,35,36^ and 2.28 to 1140.79 Darcy for gelatin methacryloyl gel^37^.

**Figure 5:**
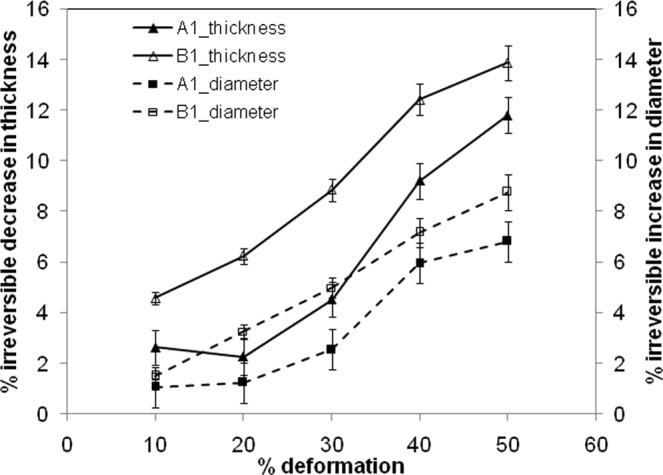
Reduction of film thickness after every deformation during the compression and reswelling of the alginate films.

The longitudinal compression is associated with lateral deformation that forms the basis for estimation of Poisson’s ratio. The cross-sectional view of the gel film without voids was imaged prior to compression, and also in the compressed state (Fig. S3 in Supplementary File) at the deformation level of 30%. The compression was conducted at 2 mm/min, and the image of the compressed film was acquired after holding the compression for 100 seconds to ensure equilibrium within the structure. The Poisson’s ratio was estimated as 0.38 ± 0.02. The Poisson’s ratios, reported by other researchers for alginate gel are within the range of 0.23 to 0.5^13,29,32,38^. However, when the extent of deformation was higher, the irreversible changes in the gel film cannot be ignored. Figure 5 presents the irreversible decrease in thickness that could not be recovered after swelling in PBS overnight. The experiments were done thrice and the error ranging from 1–8% is shown in the plot. The irreversible reduction in thickness was associated with increase in diameter of the film, shown as the secondary axis in Fig. 5. The presence of voids resulted in slightly greater changes in these two dimensions. This may also be underscored that even after the squeezing to one fifth of its original thickness, the gel film could be reswelled to two-third of its original thickness. The reswelled films showed stress-strain behaviour very similar to that of the gel film with no prior compression. As such, the deformation to this extent is merely of academic interest. The typical deformations caused by *in vivo* stresses are restricted to much smaller values^9,31,38^ e.g. 30% deformation for cartilage^39^, and 2% deformation for bone^40^. When the macrovoids were embedded in the gel structure, the irreversible changes were found to be slightly higher. This is understandably due to absence of gel network in the void zone that otherwise would have recoiled at the time of re-swelling phase.

**Figure 6:**
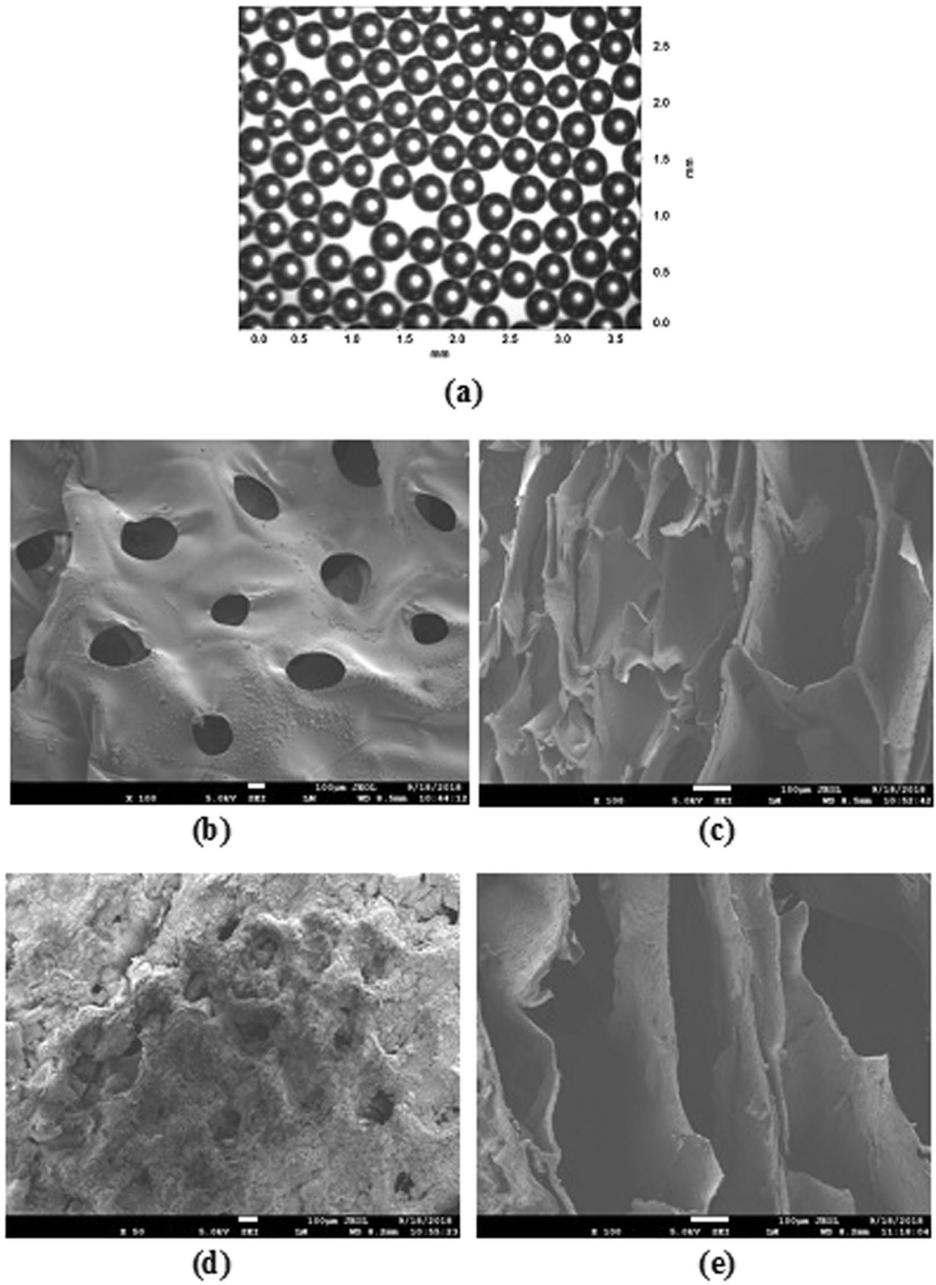
Images of alginate film (tagged as B1) (**a**) showing bubbles prior to gelation; (**b**) top view, (**c**) cross-sectional view of dry film without any deformation, and (**d**) top view, and (**e**) cross-sectional view after 70% deformation.

This aspect of permanent damage may further be explored from the SEM images (Fig. 6). The first image (Fig. 6a) was obtained under digital camera with objective immediately after bubbling in polymeric solution. The top view shows the monolayer of bubbles in the polymer film of 0.3 mm as diameter. Figures 6b,c show the top view and cross-sectional view of the lyophilized gel film with embedded voids under scanning electron microscope prior to any compression. The other set of images (Fig. 6d,e) was acquired after compressing the film to 70% deformation, followed by withdrawal of compression and freeze-drying of the film. The cross-sectional SEM images were acquired with an alignment, where the direction of compression was from left to right. The cross-sectional views show stacks in the gel network with lateral elements between them. Upon high compressive deformation, the lateral elements were partially lost. Perhaps as a consequence, the stacks did not fully recoil to their original positions after the compressive load was removed. The top views indicate that the mutual alignment of macrovoids did not change after compression and reswelling. Only the surface around the voids appears somewhat rumpled. This concurs with the understanding that the irreversible flattening of the gel film was associated with localized changes and ruptures within the gel matrix around voids.

**Figure 7:**
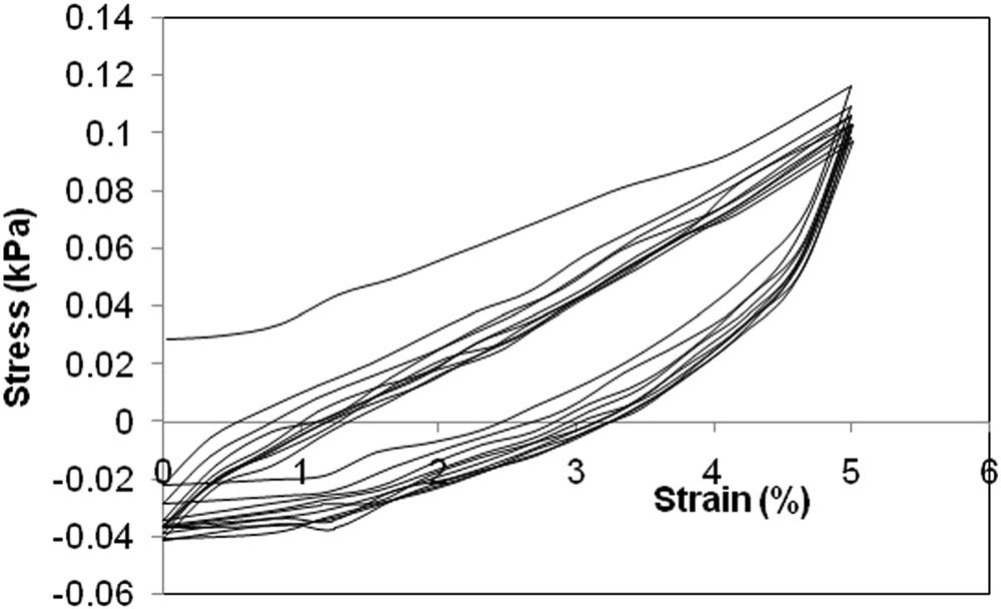
Cyclic compression of alginate film (tag no. B3), when deformed upto 5% of initial thickness at a speed rate of 2 mm/min.

This article so far was focused on building-up of compressive stress, and studying the changes in the gel structure, followed by reswelling in PBS buffer in uncompressed state overnight. The *in vivo* applications subject a gel structure to compression-decompression in cycles (Tests 3 and 4 in Fig. S2 of Supplementary File). Figure 7 shows the stress-strain response for 10 such consecutive cycles at deformation rate of 2 mm/min. The cycle started with the compression phase up to 5% deformation. Subsequently, the gel film was decompressed till the compression platen rolled back to its original position, completing one cycle of stress-strain plot. Figure 7 shows that during the decompression phase, the stress became zero at a finite strain of 3.2%. This intercept on the deformation axis can be interpreted as residual strain or stain-hysteresis for this cycle, since sufficient decompression time was not provided for the stress in the gel skeleton and the pore pressure to equilibrate over the entire structure. The decompression rate was not slow enough for the stresses in the gel structure to equilibrate, leaving a residual deformation that did not go away immediately at the end of decompression phase of the cycle. However, due to significant adhesion between gel and the compression platens, the gel was not detached from upper platen. This led to tensile stress within the gel structure till sufficient time was allowed at the end of decompression for the gel structure to completely equilibrate to its initial thickness. As such, this negative compressive stress was not given much significance by some of the earlier researchers^9,41,42^, and the cycle was reported right to the point where the compressive stress dropped to zero.

**Figure 8:**
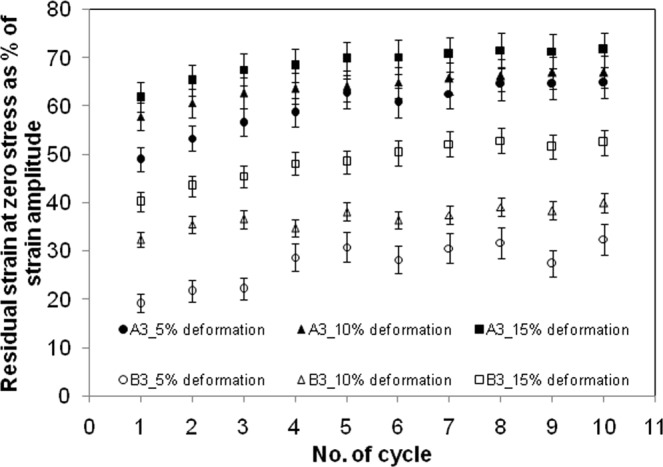
Comparison of strain at zero stress during unloading for films with tag A3 and B3 at a constant deformation rate of 2 mm/min.

**Figure 9:**
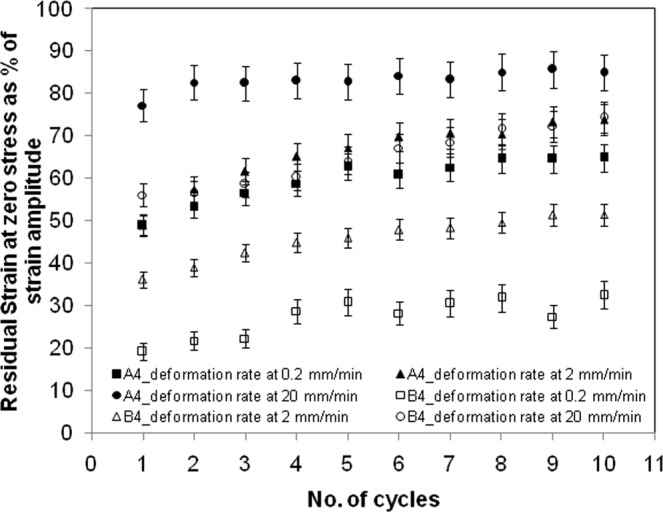
Comparison of strain at zero stress during unloading for films with tag A4 and B4 at a fixed deformation of 10%.

During the decompression phase of the stress cycle, the strain at which the stress became zero is referred here as the residual strain. This value gives a measure of hysteresis. The normalized residual strain is defined as the ratio of this strain at zero stress to the peak strain in that stress cycle. Figures 8 and 9 show this normalized residual strain, increasing moderately with progression of stress cycles. The experiments on cyclic compression were done thrice and the error ranging from 3–9% is shown in the plot. The buildup of residual strain was due to lack of sufficient time for relaxation of the crosslinked gel network. The residual strain was much smaller, when macrovoids were present. Understandably, the macrovoids presented pathways for faster equilibration of pore pressure. Also, the local deformation over the inner surface of the voids helped in equilibration of skeleton-stress at a faster rate. The increase in deformation rate has obvious effect of increase in hysteresis due to lack of time for relaxation. A lower amplitude of stress cycle gave smaller hysteresis, though the stress-reheal process slowed down as the equilibrium was approached. This is evident from substantial residual strain at small deformation.

**Figure 10:**
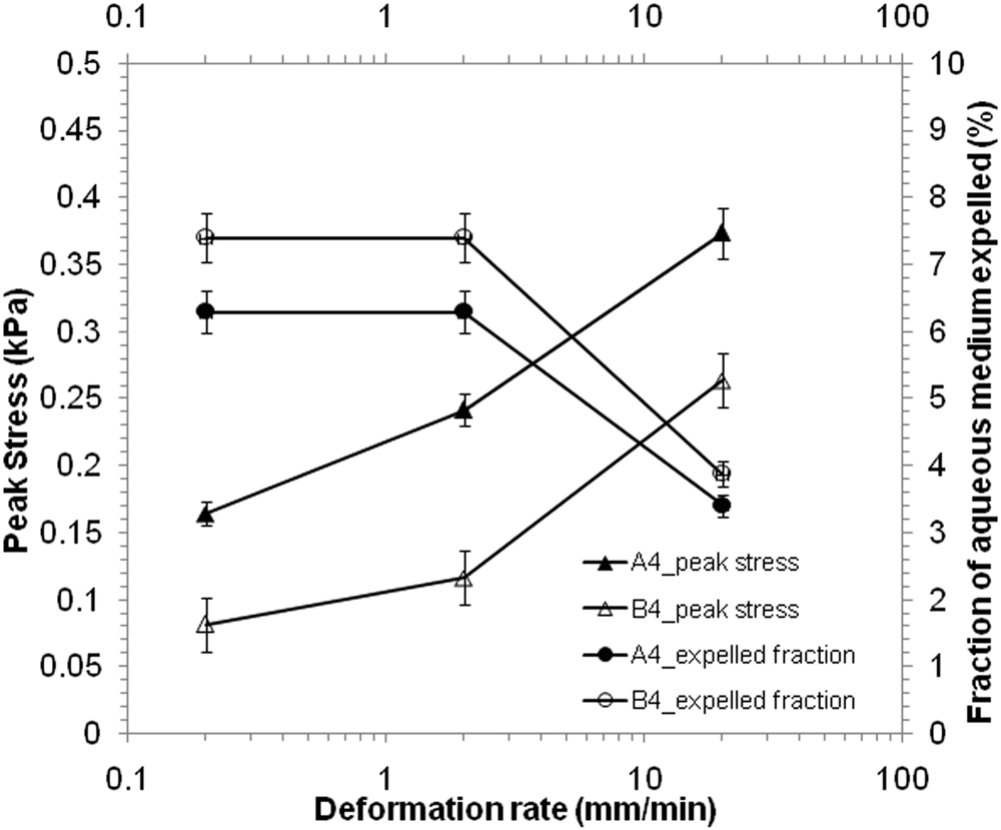
Peak stress and amount of water expelled after compression test at a fixed deformation of 5% and varying speed rate.

Figure 10 shows the peak compressive stress at the end of the cycles, conducted at different deformation rates. The increase in peak stress with the deformation rate is consistent with the observations in Fig. 1. The fraction of in-place water that was expelled during the compression phase of the cycle and reabsorbed during the decompression phase are also plotted in this figure using the option of secondary axis. Understandably, the gel structure got less time for the stress to equilibrate over the entire structure and also with the pore pressure, when the gel film was deformed at a faster rate. This resulted in less draining of aqueous phase before the decompression was set in. The presence of voids enabled faster equilibration of skeleton stress through local deformation within voids, and also by easier transport of aqueous phase through macrovoids, thereby reducing the peak stress to almost half. The greater volume was expelled at the same deformation rate, when voids were present. The deformation rate used in this study is 0.2, 2 and 2 mm/min, equivalent to the strain rate of 0.001, 0.01, 0.1 s^−1^. The deformation rate for bone^9^ and articular cartilage^39^ in the range of 0.001 to 0.1 s^−1^ were considered in previous studies. The experiments on expulsion of aqueous phase during cyclic compression were done thrice and the error ranging from 2–8% is shown in the plots.

**Figure 11:**
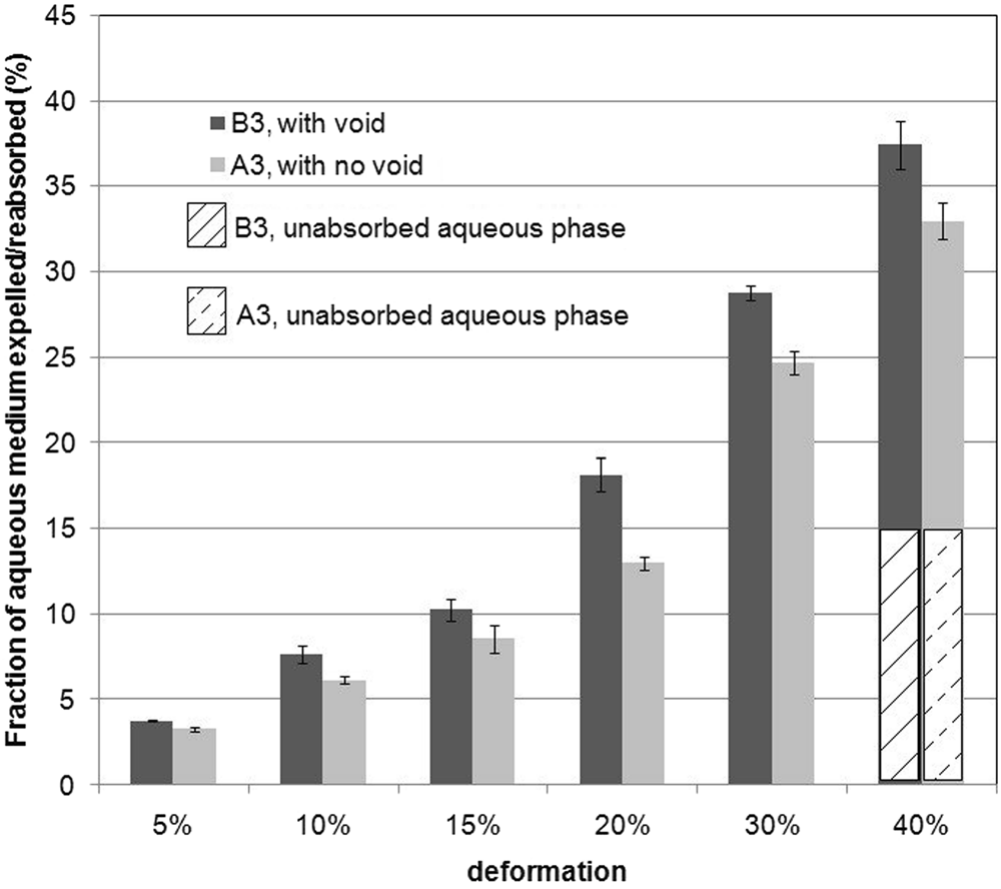
Amount of water expelled and subsequently absorbed reabsorbed during compression-decompression cycles on films A3 and B3 at a deformation rate of 2 mm/min.

In each stress cycle, the decompression was continued to the point of zero strain, to check whether the entire expelled volume could be reabsorbed. For the composition and geometry, considered here, the entire expelled fluid got reabsorbed, as long as the amplitude did not exceed 30% deformation, even for deformation rate of 2 mm/min (Fig. 11). For these cases, the negative stress at the end of the cycle (as in Fig. 7) was consistently observed. However, no detachment of upper plate from the gel film was observed, indicating a higher adhesive strength at the compression plate than the negative stress, referred above. The experiments were done thrice and the error ranging from 3–8% is shown in the plots. When the deformation amplitude exceeded 40%, a clear detachment of the upper platen from the gel structure was observed. At 40% deformation, almost half of the expelled aqueous phase was not reabsorbed over the decompression time frame, considered in this study. The unabsorbed fraction at the end of compression cycle is shown in Fig. 11 as hatched area. As evident from the earlier results of stage-wise compression, the reswelling in PBS buffer overnight would have resulted in reabsorption to the extent of 90% of the original volume.

Therefore, the above results throw light on how alginate gel structure behaves under compressive loading, and how the presence of macrovoids affects the behaviour. The voids were dispersed uniformly across the structure, using a fluidic arrangement. The macrovoids are known to help in colonization of regenerated tissue cells, and “cushion” large deformation with build-up of small stress. The cyclic compression-decompression is expected *in vivo* due to various reasons, ranging from blood flow to muscle movement, where the amplitude is small^43^. An example of *in vivo* stress, apart from movement in muscles is due to blood flow. Mean blood pressure in arteries is greater than the surrounding tissue by approximately 13 kPa at the level of heart. This value is higher at a level, hydrostatically below the position of heart, and is smaller at a position above the level of heart. This constitutes a compressive stress, changing cylindrically with blood flow in the tissue surrounding the blood vessel. On the other hand, excessive build-up of compressive stress could be highly detrimental. Extraneural compression pressure as low as 2.5 kPa can decrease intraneural microvascular flow, and a pressure of 4 kPa can impair axonal transport leading to endoneurial edema formation, and finally axonal degeneration. Skeletal muscles, when activated to contract by stimuli cannot sustain intramascular pressure greater than 4 kPa for 8 hours without causing muscle-fibre atrophy, spitting, necrosis, and other derangements. Cartilaginous end plates in the intervertebral disc can withstand compression typically upto 5.2 ± 1.8 kN depending on vertebral cross-sectional area and bone density. Therefore, the sensitivity of different body elements surrounding a gel scaffold demands that additional stresses arising from compressive deformation be absorbed by the gel scaffold^44^.

In this study, the alginate gel without voids registered a stress of 2 kPa at 40% deformation, and this value could be cut down by 20% through introduction of voids. The previous reports suggested ultimate stress of alginate^45^ in the range of 2–38 kPa, gelatin gel crosslinked with glutaraldehyde^46^ in the range of 1.3 to 5.1 MPa, and chitosan^47,48^ in the range 20 to 76 MPa. In this research, the gel structure is subjected to compressive loading of varied amplitudes and number of cycles at different deformation rates. The gel structure, being a viscoelastic polymer network with trapped water constituting more than 95% of the total volume, the response to the cyclic loading was not obvious. On one hand, the gel structure tends to accommodate the deformation by expelling the trapped water from the pore space and/or from the macrovoids. At the same time, the crosslinked skeleton responds to the compressive stresses like any other viscoelastic material. Both the responses are reversed when the decompression part of the cycle sets in. This study systematically reviewed the responses of the gel structure by measuring the water expulsion and reabsorption at each cycle vis-a-vis the stress-strain curve. During compression, the voids provided hydraulic pathways for the movement of aqueous phase, thereby enabling better equilibration of skeleton stress with the pore pressure across the structure. Also, the voids provided local sites for lateral stretching. Thus, voids helped in quicker equilibration of stress, and greater release of aqueous phase during compression. This resulted in smaller build-up of compressive stress during stress cycles that is advantageous in countering *in vivo* stresses, and in providing lubrication.

## Conclusions

In this article, the alginate gel film was studied under repeated cycles of compression and decompression. A monolayer of macrovoids was introduced in regular alignment within the gel structure using a fluidic arrangement. The photographs were analyzed to ascertain the lateral deformation at various stages of linear compression. The SEM images before and after deformation were reviewed to determine the changes in the microstructure from the cross-sectional views, and from the topographical changes around the macrovoids. The expulsion and reabsorption of aqueous phase upon compression and decompression respectively were monitored.

A comparison of the stress responses from the relaxation test show that both the dry gel film and swelled film without voids underwent stress relaxation, when the strain was held constant with time. The peak stress was much higher for the dry film. The peak stress was reached much faster in dry film for the same deformation rate. The relaxation was also much faster, indicating the influence of aqueous phase on the stress response. When voids were present, the build-up of compressive stress was less, under monotonously increasing compression and the expulsion of aqueous phase was more. The accommodation of lateral deformation locally within the macrovoids, and easier release of pore pressure by transport of aqueous phase through the network of macrovoids are hypothesized for these favourable characteristics. A simplified momentum balance equation was drawn along with the volumetric balance to estimate the average permeability of the gel structure. The permeability decreased with compressive loading, and the reason is attributed to the compaction of gel strands that constricted the expulsion pathways. The presence of voids resulted in less restriction on the flow through gel structure, thereby increasing the permeability, particularly at smaller deformation. The aqueous phase, expelled during compression was fully reabsorbed within the time frame of decompression that was conducted at 2 mm/min. However, the reabsorption was not complete at this decompression rate, when the initial compression was increased to 40% deformation. Also, beyond this level of deformation, a permanent flattening i.e. irreversible reduction in thickness and increase in diameter became considerable. However, such level of deformation does not bear much of significance, as the typical upper limit for articular cartilage^39^ for different forms of movement was considered as 30%. The irreversible reduction in thickness was slightly more when voids were present. The hysteresis in stress cycles showed significant decrease, when voids were present. The peak stress got increased, and expulsion of aqueous phase got decreased, with increase in deformation rate for both with-void and without-void films. The chemical analysis of expelled fluid did not show evidence of any stress-induced disintegration of gel film.

Therefore, this research shows promise of embedded macrovoids in (i) reducing the compressive stresses within gel structure substantially, (ii) quicker re-heal of residual stresses (and thus reducing hysteresis under repeated stress cycles), (iii) larger expulsion of pore fluid upon compression (and thus offering lubrication for use in joints), and (iv) faster reabsorption of expelled fluid upon decompression in repeated cycles. Even after hardest compression, the macrovoids and their mutual alignment were retained in alginate gel. These structural benefits of macrovoids in biopolymer gel are over and above the other potential advantages e.g., higher uptake and release of bioactive species, and extra space for cell colonization. Microfluidic method of void embedding enabled maintenance of high degree of uniformity across the gel structure.

## Material and Methods

The alginate gel was made by crosslinking the aqueous suspension of sodium alginate. Here, calcium chloride is the crosslinker. As a surfactant, Pluronic F127 was added to the aqueous suspension of biopolymer. The release and reabsorption studies were conducted in phosphate buffer saline (PBS), which is essentially a mixture of sodium chloride (NaCl), potassium chloride (KCl), disodium phosphate (Na_2_HPO_4_) and potassium dihydrogenphosphate (KH_2_PO_4_) in ultrapure water (milli-Q). Sodium alginate of low viscosity type (100 to 300 cP) and Pluronic F-127 were supplied by the Sigma Aldrich Co., USA. Calcium chloride (CaCl_2_), sodium chloride (NaCl), potassium chloride (KCl), disodium phosphate (Na_2_HPO_4_) and potassium dihydrogenphosphate (KH_2_PO_4_) of analytical grade were supplied by Merck Specialities Pvt. Ltd., India. Ellenbarrie Industrial gases Ltd., India supplied the nitrogen gas that was utilized in bubble formation.

Sodium alginate (4 wt%) in ultrapure water (milli-Q) was stirred at a speed of 400 rpm for 24 hours. Pluronic F – 127 (4 wt%) was dissolved in milli-Q water by refrigerating the mixture at a temperature of 10 °C. The pluronic solution was added to the alginate suspension and the mixture was stirred for 4 hours. Subsequently, the mixture was flowed alongside the nitrogen gas in the “orifice in throat device” to generate bubbles. Further, details of the flow device are in Patra *et al*.^25^. The aqueous phase was injected into the device using a syringe pump (Harvard Apparatus, U.S.A). The gas phase was injected using a mass flow controller (Alicat Scientific, U.S.A). The liquid and the gas flow rates were precisely maintained at 8 mL/min and 1 mL/min respectively^18,29,33^. The stretching and squeezing of the gas string at the tip of the fluidic device resulted in breaking of gas string into small and uniform bubbles. After collection of bubbles in a petridish, the polymer in the aqueous phase was crosslinked through addition of 4 wt% CaCl_2_ solution in drops. The diameter and thicknesses of the films are given in Table 1. A shelf-type lyophilizer unit was used for removal of moisture from the film. The lyophilisation process was composed of three phases. In the first phase (freezing), the temperature of the film was reduced to −30 °C. In the second phase (sublimation), the vacuum of 0.09 mbar (absolute) was imposed on the film, as the temperature was increased to 0 °C. The increase of temperature from −30 °C followed a ramp of 10 °C/60 min, and a soak at −20 °C, −10 °C, and 0 °C for 45 minutes. In the third phase (secondary drying), the temperature was increased all the way to 40 °C through a similar ramp and soak sequence^29^. The vacuum was maintained throughout the secondary drying process. The films with individual tags are listed in Table 1. The alginate films with voids are identified as B1, B2, B3, and B4. The remaining films did not contain any void. Also, the numbers in the tag indicates the respective tests (Fig. S2, in the Supplementary File) performed on the films.

The dried films were observed under a scanning electron microscope (JEOL JSM5800 from JEOL, Japan), and the digital images were acquired. The sample for imaging was gold-sputtered after mounting it on a metal grid. Also, the image of the film was obtained immediately after collection on petridish using a regular microscope and Davis Software from LaVision GmbH.

The dry gel films were soaked overnight in PBS buffer solution (pH 7.4). Subsequently, the films were placed in a transparent cylindrical holder, made of perspex (Fig. S1, in Supplementary File). This holder was specially designed to study the changes in the gel, and to measure the volume of expelled or reabsorbed solution, as the film was subjected compressive load. A glass capillary of 2 mm OD was bent in the form of U-shape, and was attached to the Perspex holder. The U-portion of the capillary tube, and also thin circumferential grove that is buried at the inner edge of the cylindrical holder were filled with PBS buffer, thus ensuring the fully soaked condition of the gel film inside the holder. The Universal Testing Machine (UTM) from Tiniun Olsen U.K. was used here for the compression studies. As the upper compression platen pressed the gel film from the top, the expelled fluid from the film pushed the liquid level up along the right limb of the U-tube. The rise in liquid level was measured using the precalibrated marks on the right limb.

The Poisson’s ratio was estimated from the front views of the film, captured using a camera with an objective from LaVision GmbH. Based on the relative positions of the plates and the diameters of film, the lateral and the longitudinal strains were estimated. These estimates were also useful in ascertaining the volume of fluid.

Figure S2 in Supplementary File shows the sequence of compressive loadings on the films. Four types of sequences (Tests 1, 2, 3, and 4) are shown here with the % deformation in y-axis. The shaded gaps between the ramps indicate overnight (12 hours) swelling in PBS buffer. The first test describes sequential compression to different level of deformation. The last two sets describe compression-decompression cycles at varying amplitude and frequency respectively. The volume of expelled, and subsequently reabsorbed aqueous phase was monitored at every stage of the experiment. Separate experiments were conducted on dry film, as well as wet film, to study stress relaxation in the gel network. In these experiments, the stress was built up fast at constant deformation rate, and then the deformation was held unchanged for 100 seconds, while the stress was monitored with time^29^.

The expelled fluid was analyzed for calcium content by atomic absorption spectrophotometry (AAS) (PerkinElmer Instruments, USA). For this purpose, the compression was conducted on film K in a separate petridish, so that the expelled fluid can be drawn without contamination by the surrounding PBS buffer. The expelled fluid was kept in tightly capped glass vial to avoid any evaporative losses. The alginate if any in the expelled fluid was dissolved by mixing with a chelator solution (0.1M EDTA and 0.2M sodium citrate). The volume ratio of expelled fluid to the chelator solution was taken as 1:50. The dissolution was ensured by sonicating the resultant mixture for 30 minutes. For comparison, the freshly prepared alginate gel was also submitted to the same dissolution protocol, and the calcium content was measured.

## Supplementary information

Supplementary Dataset 1
