# Supplementary material for: Mechanical behaviour of alginate film with embedded voids under compression-decompression cycles
Source: Sci Rep. 2019 Sep 13;9:13193. doi: 10.1038/s41598-019-49589-w (PMC6744475; doi:10.1038/s41598-019-49589-w)
Supplement: Supplementary file 1 — Supplementary Dataset 1 [file 41598_2019_49589_MOESM1_ESM.docx]

Supplementary file

**Mechanical behaviour of alginate film with embedded voids under compression-decompression cycles**

Authors: Arindam Banerjee, Somenath Ganguly


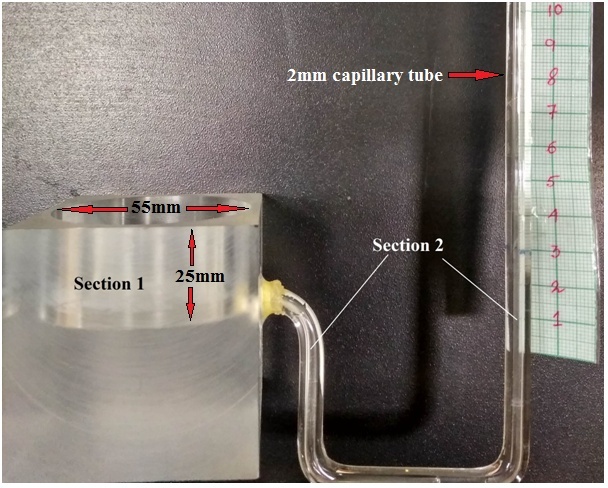


**Figure S1: Perspex device where the films are kept in section 1 and during mechanical testing the amount of liquid expelled is measured in section 2.**


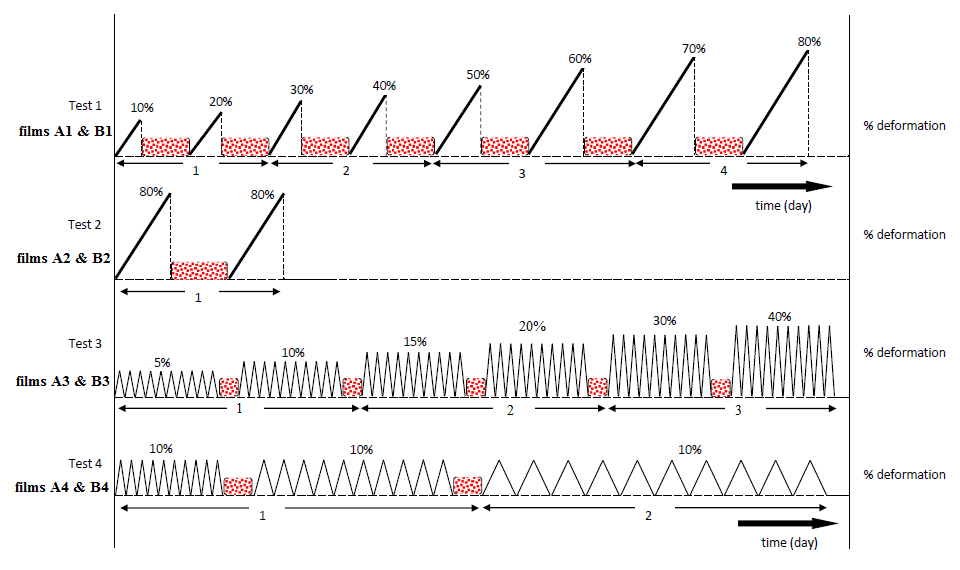


**Figure S2: Sequence of compressive and cyclic deformation on the alginate films.**

**
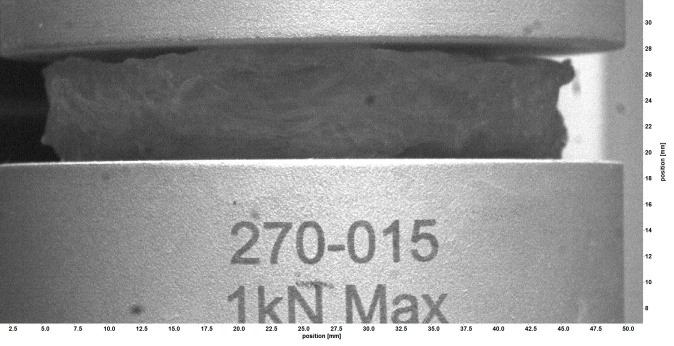

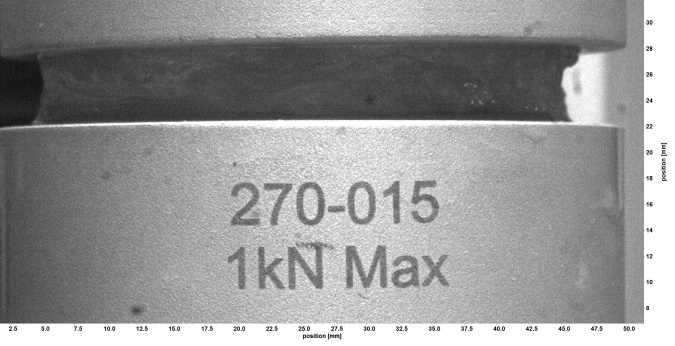
**

**(a) (b)**

**Figure S3: Images of alginate (film A) (a) initially and (b) after deforming 30% to determine the Poisson’s ration.**
